# Estimation of Genetic Parameters for Milk Production Rate and Its Stability in Holstein Population

**DOI:** 10.3390/ani14192761

**Published:** 2024-09-24

**Authors:** Hailiang Zhang, Qing Gao, Ao Wang, Zichen Wang, Yan Liang, Mengling Guo, Yongjiang Mao, Yachun Wang

**Affiliations:** 1 Laboratory of Animal Genetics, Breeding and Reproduction, Ministry of Agriculture of China, National Engineering Laboratory of Animal Breeding, State Key Laboratory of Farm Animal Biotech Breeding, College of Animal Science and Technology, China Agricultural University, Beijing 100193, China; zhl108@cau.edu.cn (H.Z.); 15096111700@163.com (Q.G.); lxxkwa@163.com (A.W.); 2 College of Animal Science and Technology, Yangzhou University, Yangzhou 225009, China; shirleywang2020@126.com (Z.W.); mz120181016@yzu.edu.cn (Y.L.); g18852720440@163.com (M.G.)

**Keywords:** milk production rate, milk yield, heritability, genetic analysis, dairy cattle

## Abstract

**Simple Summary:**

This study aimed to estimate the phenotypic and genetic parameters of milk production rate (MPR) traits in Holstein cattle. The MPR, a metric for evaluating a cow’s milk secretion per hour, was calculated using milk yield and milking interval data from 4760 cows. This study defined four milk yield and six MPR traits, and used the MIXED procedure to assess the effects of parity, season, and lactation stage on these traits. Significant effects of these non-genetic factors were found for both milk yield and MPR traits. Heritability estimates for milk yield and MPR traits were high, ranging from 0.25 to 0.39, while the stability of MPR had low heritability (0.04 to 0.05). This study concluded that MPR is a valuable trait for dairy breeding, providing new insights for herd management and genetic selection to improve dairy cattle production performance.

**Abstract:**

Milk production rate (MPR) refers to the rate of milk secretion per hour (kg/h), calculated from the harvested milk yield and milking interval, and it is considered an appropriate measure to evaluate the production potential of cows. The objective of this study was to estimate the phenotypic and genetic parameters of milk production rate traits. In this study, the milking records of 4760 Holstein cows were collected, and four milk yield traits and six milk production rate traits were defined. The MIXED procedure was used to detect the impacts of non-genetic effects on milk yield and milk production rate traits, including parity, measured season and lactation stage. Variance and covariance components for milk yield and milk production rate traits were estimated using a univariate linear repeatability model. Parity, measurement season and lactation stage had significant effects (*p* < 0.01) on milk yield, milk production rate and its stability. Milk yield and milk production traits had high heritability, and ranged from 0.25 to 0.39. The stability of milk production rate had low heritability (0.04~0.05). Milk production rate is beneficial for the devolving novel trait in dairy breeding and provides new insights for herd management and genetic selection of production performance of dairy cattle.

## 1. Introduction

In dairy cattle breeding, milk production is the most important economic trait with the largest weight in breeding goals around the world. Since the turn of the 20th century, the selection for milk production has been going on for more than 100 years, and great genetic gains have been achieved by dairy breeders [1,2]. Generally, dairy associations from various countries collect test-day milk yield records to perform genetic evaluation for milk production traits, such as daily milk yield and 305d milk yield traits [3]. Measurements on daily records are very effective for the selection for milk production traits, and large genetic gains have been obtained by intensive genetic selection [1]. However, this method loses a lot of detailed information about individual lactation curves. 

With the application of electronic milk measurement and automated milking systems, a large amount of accurate longitudinal data for milk performance provided new opportunities for phenotyping for production. For example, milk loss and milk resilience, developed on the basis of continuous daily milk yield records, have become a hotspot in dairy breeding research [4,5]. In addition to high-density yield records, the milking interval can be recorded accurately to the minute. Milk production rate was defined as the rate of milk secretion per hour (kg/h), calculated from the harvested milk yield and milking interval [6]. By integrating the records of milk yield and milking interval, the milk production rate would be considered an appropriate metric to evaluate the production potential of cows. For example, milk production rate has been used to investigate the effect of incomplete milking [6,7], and to analyze its association with clinical mastitis [8,9]. Penry et al. (2018) reported the population characteristics for standard deviations of quarter milk production rate [10]. Compared with daily milk yield, MPR can accurately reflect the production performance of dairy cows, which provided the most direct and valuable information for management and reminded farm managers to find out the causes of milk production fluctuations. Furthermore, the stability of milk production rate partly reflects the resilience of dairy cows to a certain extent. The standard deviation and the range of MPR can access the stability of MPR of different shifts within one day, which can become new indicators for resilience breeding of dairy cows.

In previous studies, the genetic parameters of milk production traits have been widely reported in the Holstein population, and with moderate-to-high heritability ranging from 0.1 to 0.4 [11,12,13]. However, there is a lack of research reporting the phenotypic and genetic characteristics of milk production rate traits. In this study, we performed phenotyping and genetic analysis to obtain the phenotypic and genetic parameters using the records from a large dairy farm, and the results of this study provide new insights for herd management and the genetic selection of production performance of dairy cattle. 

## 2. Materials and Methods

### 2.1. Raw Data

In this study, the milking records of 4760 Holstein cows from a large dairy farm in Jiangsu (China) were collected from January 2018 to July 2019. In this dairy farm, all lactating cows had ad libitum access to water and TMP (total mixed ration) and were milked three times a day in a parlor system. Raw data included 619,208 milking shifts from 4760 Holstein cows, including individual information (birth date, parity, and calving date) and milking information (milking date, milk yield and milking recognition time for each shift).

The raw pedigree was provided by the dairy farm and the Dairy Association of China (Beijing). Each animal with the phenotype was traced back as many generations as possible. The final pedigree used for genetic analysis consisted of 7190 females and 623 males.

### 2.2. Data Editing and Trait Definition

During data processing, the records without parity information, milking date or calving date were firstly removed, as were the records with a DIM (days in milk) of more than 365 days. In addition, only the records with a milk yield between 2 and 30 kg were retained. After data processing, a total of 533,845 milking records from 4529 cows were used for further analysis. 

In this study, the milking interval was calculated for each shift based on the milking recognition time, and it refers to the interval between the milking identification time of the current shift and that of the last shift. The daily interval was the sum of the milking intervals for three shifts. The milk production rate for each milking shift was calculated according to the milk production by the milking interval of the corresponding milking shift, including the milk production rate in the morning (MPR-Morn), noon (MPR-Noon) and night shifts (MPR-Night). Similarly, the daily milk production rate (DMPR) was calculated according to the daily milk production by the daily milking interval. To evaluate the stability of the milk production rate within a day, the standard deviation (MPR-SD) and range (MPR-R) of the milk production rate within a day were calculated based on MPR-Morn, MPR-Noon and MPR-Night. Furthermore, four milk yield traits were analyzed in this study, including the daily milk yield (DMY), and the milk yield in the morning (MY-Morn), noon (MY-Noon) and night shifts (MY-Night). The abbreviations and definitions of all ten traits analyzed in this study are shown in Table 1. 

### 2.3. Statistical Analysis

The MIXED procedure of SAS software (version 9.1; SAS Institute, 2004) was used to detect the effects of non-genetic effects on milk yield and milk production rate traits, including parity, measured season and lactation stage. In addition, an individual random effect was included in the mixed model. The Bonferroni *t*-test was employed to perform multiple comparisons between different levels for each non-genetic effect. In this study, parities of cows were divided into five levels, 1, 2, 3, 4, and ≥5, where the fifth level included cows from parity 5 to parity 11; lactation stages were divided into 5 levels, including 1–44 d, 45–99 d, 100–199 d, 200–305 d, and >305 d. According to the climatic characteristics of Jiangsu (China), the test season can be divided into four categories, including spring (March, April and May), summer (June, July and August), autumn (September, October and November) and winter (December, January and February).

Variance and covariance components for milk yield and milk production rate traits were estimated using the average information restricted maximum likelihood algorithm implemented in the DMU software (Version 6) [14]. Heritability and repeatability were estimated using a single-trait linear repeatability model for each trait. Genetic correlations between the stability of the milk production rate and milk production rate, and milk yield traits were estimated using a bivariate linear repeatability model. The model fitted for milk yield and milk production rate traits was as follows:

(1) $$Y_{ijkl}=SEASON_{i}+LACT_{j}+STAGE_{k}+a_{ijkl}+pe_{ijkl}+e_{ijkl}$$

where *Y* is the vector of phenotypes for DMY, MY-Morn, MY-Noon, MY-Night, DMPR, MPR-Morn, MPR-Noon, MPR-Night, MPR-SD and MPR-R; $SEASON_{i}$ is a vector of fixed effect of testing season (*i* = 1, 2, …, 4, representing spring, summer, fall and winter); $LACT_{j}$ is a vector of the fixed effect of parity (*j* = 1, 2, …, 5, representing parity 1, 2, 3, 4, and 5 and above); $STAGE_{k}$ is a vector of the fixed effect of lactation stage (*k* = 1, 2, …, 5, representing 5 lactation stages as described above). $a_{ijkl}$ is a random additive genetic effect, $a\;\sim \;\left(0\;,\;A\sigma_{a}^{2}\;\right)$; $pe_{ijkl}$ is a random permanent environmental effect,$\;pe\;\sim \;\left(0\;,\;I\sigma_{pe}^{2}\;\right)$; $e_{ijkl}$ is a random residual effect, $e\;\sim \;\left(0\;,\;I\sigma_{e}^{2}\;\right)$; where **A** is the matrix of additive genetic relationships constructed from the pedigree, $\sigma_{a}^{2}$ is the additive genetic variance, **I** is the identity matrix, $\sigma_{pe}^{2}$ is the permanent environmental variance, and $\sigma_{e}^{2}$ is the residual variance.

Univariate analyses were performed to estimate the heritability ($h^{2}$), which was defined as $h^{2}=\frac{\sigma_{g}^{2}}{\sigma_{g}^{2}+\sigma_{pe}^{2}+\sigma_{e}^{2}}$ (Model 2), and repeatability (t) was defined as $t=\frac{\sigma_{g+}^{2}\sigma_{pe}^{2}}{\sigma_{g}^{2}+\sigma_{pe}^{2}+\sigma_{e}^{2}}$ (Model 3), where $\sigma_{g}^{2}$ is the additive genetic variance, $\sigma_{pe}^{2}$ is the permanent environmental variance, and $\sigma_{e}^{2}$ is the residual variance in the corresponding trait.

## 3. Results

### 3.1. Descriptive Statistics

The descriptive statistics for the milk yield traits and milk production rate traits are presented in Table 2. In this study, the average daily milk yield ranged from 6 to 79.9 kg, with an average of 33.87 kg. Among the three milking shifts, the average milk yield in each milking shift ranged from 11.12 (night milking shift) to 11.44 kg (noon milking shift). 

**Figure 1:**
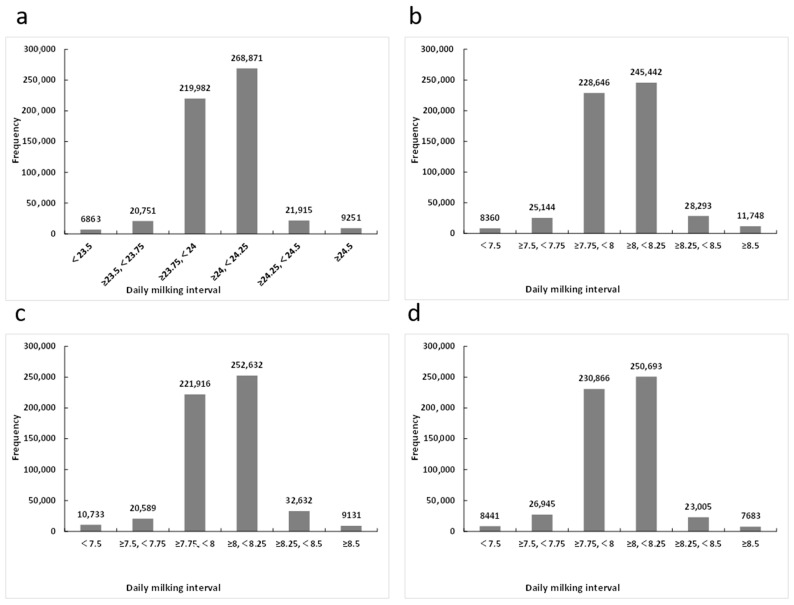
The distribution of milking interval in daily (**a**), morning (**b**), noon (**c**) and night (**d**) shifts.

The distribution of the daily milking interval and the milking interval in the morning, noon and night milking shifts is shown in Figure 1. The daily milking interval was mostly concentrated in the range of 23.75 to 24.25 h, and the milking intervals in the three milking shifts were concentrated in the range of 7.75 to 8.25 h. Furthermore, the milk production rate ranged from 1.40 (Night milking shift) to 1.44 kg/h (Noon milking shift), and the daily milk production rate was 1.42 kg/h. Among the three milking shifts within a day, the difference in milk production rate reached 0.37 kg/h for the same individual, and the standard deviation of the milk production rate among the three shifts per day was 0.16.

### 3.2. Impacts of Nongenetic Effects 

In this study, parity, measurement season and lactation stage had significant effects (*p* < 0.01) on milk yield traits. Least squares mean estimates of different levels and multiple comparisons based on Bonferroni t-corrected are presented in Table 3. As presented in Table 3, the cows in lactation 4 had the highest milk yield, and there were highly significant differences between the five parity levels. In terms of lactation stage, the milk yield of cows firstly increased and then gradually decreased with the change in lactation stage. Among four seasons, the cows had the highest milk yield in winter and the lowest in summer. Furthermore, the similar change trends of non-genetic effects on milk yield were observed between daily milk yield (DMY) and shift milk yield traits (MY-Morn, MY-Noon and MY-Night).

Parity, lactation stage, and season had highly significant effects on milk production rate traits; the least squares mean estimates of these three factors are presented in Table 4. In addition, similar trends of the milk production rate with a change in parity, lactation stage, season were found for daily, and morning, noon and night milking shifts. Among the five parity levels, cows in parity 4 had the highest milk production (1.54~1.58 kg/h), which was significantly higher than that of other parity levels (*p* < 0.01). With the change in lactation stage, the milk production rate first increased from 1.59~1.62 kg/h (DIM 1~44 d) to 1.71~1.76 kg/h (DIM 45~99 d) and then decreased to 1.11~1.16 kg/h (DIM > 365 d). Among the four seasons, the milk production rate was highest in winter (1.54~1.61 kg/h) and lowest in summer (1.31~1.32 kg/h). Furthermore, a larger difference among seasons was observed in winter (0.07 kg/h) and spring (0.04 kg/h) than in summer (0.01 kg/h) and fall (0.01 kg/h) among the three milking shifts within a day.

The effects of parity and lactation stage on the stability of milk production rate within one day are presented in Table 5. As shown in Table 5, the variation in milk production rate between the three milking shifts was significantly larger in the parities of 4 and above than that in other parities. Within a lactation period, the cows in early lactation (DIM less than 100 d) had a relatively large variation in the milk production rate within one day. In terms of season, cows in summer had the largest variation in milk production rate within one day.

### 3.3. Genetic Parameters

The variance components, heritability and repeatability for milk yield and milk production rate traits are presented in Table 6. Milk yield traits (DMY, MY-Morn, MY-Noon and MY-Night) and milk production traits (DMPR, DMPR-Morn, DMPR-Noon and DMPR-Night) had high heritability, ranging from 0.25 (Noon-MY) to 0.39 (DMY). However, the stability of the milk production rate had low heritability (0.04~0.05). The genetic correlations of the stability of milk production rate with the milk production rate and milk yield traits are presented in Table 7. High genetic correlations were observed, ranging from 0.678 (MSR-SD and MY-Noon) to 0.726 (MSR-Night and MSR-R).

## 4. Discussion

In this study, each cow was milked three times a day, and the shift in milk yield (milk yield each milking) of the current population was within the reported range (8.2~14.0 kg) of Holstein cows in previous studies [8,15,16]. Furthermore, the average milk production rate in each milking shift ranged from 1.40 to 1.44 kg/h, with an average of 1.42 kg/h for daily milk production rate in Holstein cattle. In a previous study, the milk production rate was widely investigated in Holstein cattle, and it ranged from 0.97 to 1.97 kg/h in Italian [16] and US [6,7,10] Holstein population. 

It was found that parity, lactation and season had significant effects on milk production rate traits. For example, the milk production rate was the lowest in summer due to the severe heat stress, and large differences (0.23~0.29 kg/h) for the milking production rate were observed for the three milking shifts among different seasons. In previous studies, the relationships between milk production rate and incomplete milking [6,7], milking frequency [7], and clinical mastitis [8] were reported in various dairy populations. Collectively, the milk production rate is sensitive to the physiological status of cows and their environment and it is a good indicator to use in herd management. In addition, the effects of parity, lactation stage and season on milk yield traits have been widely reported in previous studies [17,18,19], which is consistent with the findings of the current study. 

Based on the continuous records by automated milking systems, Elgersma et al. [4] indicated that fluctuations in milk yield can be used as a resilience indicator for breeding healthy cows. In the current study, the standard deviation (MPR-SD) and the range (MPR-R) of milk production rate within a day were defined and analyzed as the resilience indicator. It was found that these two resilience indicators also were also sensitive to the parity, lactation and season effects. Specifically, the cows in later parity and during the early lactation period had a poor performance in resilience, which is similar to the findings of Poppe et al. [20] and Chen et al. [21]. In addition, in summer, the cows had the worst resilience among the four seasons. In the study by Gantner et al. [22], it was found that high-yield Holstein cows were more susceptible to heat stress than low-yield cows, and heat stress is one of the most common sources of stress in dairy cows. 

In this study, the genetic parameters were estimated for daily milk yield and milk yield in each milking shift. Moderate-to-high heritability (0.25~0.39) was found for DMY, MY-Morn, MY-Noon, and MY-Night, which is similar to the heritability estimates for milk yield traits with other definitions [3,23]. Furthermore, heritability and repeatability estimates were obtained for milk production rate traits, and moderate-to-high heritability and repeatability were also found for milk production rate traits (DMPR, MPR-Morn, MPR-Noon, and MPR-Night). There was a similar genetic architecture between milk yield and milk production traits. However, the stability of the milk production rate had low heritability (0.04~0.05). In previous studies [5,21,24], the characteristic of low heritability for resilience indicators was reported, and most of the estimates were lower than 0.1, which is consistent with the findings of the current study.

Although this study provides valuable insights into the genetic characteristics of milk production rate (MPR) in Holstein cattle, the relatively small data sample size of 4760 cows limits the representativeness of the estimates. The results may not be fully generalizable to the broader Chinese Holstein population or to Holstein populations in other regions due to differences in genetic background and environmental conditions. Future studies with larger and more diverse populations are needed to obtain more representative genetic parameter estimates to ensure a broader applicability of the results. MPR provides insight into production efficiency and consistency across milking shifts, while MPR stability provides a measure of resilience under varying milking shifts. The introduction of the milk production rate (MPR) expands the data sources available for selecting milk yield traits, moving away from relying solely on daily milk yield data from DHI systems. By integrating these traits into breeding indices, it is possible to select cows that produce milk consistently and efficiently. The milk production rate and its stability are heritable; so, these can be used in dairy breeding.

## 5. Conclusions

In this study, the daily milk yield ranged from 6 to 79.9 kg, with an average of 33.87 kg, and the milk production rate ranged from 1.40 (night milking shift) to 1.44 kg/h (noon milking shift), and the daily milk production rate was 1.42 kg/h. Parity, lactation stage and season had significant effects on milk yield, milk production rate traits and their stability. The milk yield traits and milk production traits had high heritability and ranged from 0.25 to 0.39. The stability of the milk production rate had low heritability (0.04~0.05). The results of this study provided a new perspective for the selection of lactation performance and an improvement in the breeding index of dairy cattle.

## Figures and Tables

**Table 1:** Definitions of milk yield and milk production rate traits in Holstein cattle.

Items	Abbreviation	Definition
Daily milk yield, kg	DMY	total milk yield in a day
Milk yield in the morning, kg	MY-Morn	milk yield in the morning milking shift
Milk yield in the noon, kg	MY-Noon	milk yield in the noon milking shift
Milk yield in the night, kg	MY-Night	milk yield in the night milking shift
Daily milk production rate, kg/h	DMPR	average milk production per hour of a day
Milk production rate in the morning, kg/h	MPR-Morn	average milk production per hour in the morning shift
Milk production rate in the noon, kg/h	MPR-Noon	average milk production per hour in the noon shift
Milk production rate in the night, kg/h	MPR-Night	average milk production per hour in the night shift
Standard deviation of milk production rate	MPR-SD	standard deviation of milk production rate among three shifts per day
Range of milk production rate, Kg/h	MPR-R	range of milk production rate among three shifts per day

**Table 2:** Descriptive statistics of milk yield and milk production rate traits in Holstein cattle.

Trait *	No. Records	Mean	SD	CV	Min	Max
DMY (kg)	533,845	33.87	10.31	30.43%	6.00	79.90
MY-Morn (kg)	533,845	11.33	3.92	34.59%	2.00	30.00
MY-Noon (kg)	533,845	11.44	3.82	33.37%	2.00	30.00
MY-Night (kg)	533,845	11.12	3.80	34.19%	2.00	30.00
DMPR (kg/h)	533,845	1.42	0.42	30.44%	0.25	3.33
MPR-Morn (kg/h)	533,845	1.43	0.49	35.00%	0.17	5.78
MPR-Noon (kg/h)	533,845	1.44	0.47	33.49%	0.23	4.70
MPR-Night (kg/h)	533,845	1.40	0.46	33.95%	0.17	3.77
MPR-SD	533,845	0.16	0.15	94.64%	0.00	2.07
MPR-R (kg/h)	533,845	0.37	0.35	93.67%	0.00	4.45

* DMY, total milk yield in a day; MY-Morn, milk yield in the morning milking shift; MY-Noon, milk yield in the noon milking shift; MY-Night, milk yield in the night milking shift; DMPR, daily milk production rate; MPR-Morn, milk production rate in morning shift; MPR-Noon, milk production rate in noon shift; MPR-Night, milk production rate in night shift; MPR-SD, standard deviation of milk production rate among three shifts per day; MPR-R, range of milk production rate among three shifts per day.

**Table 3:** Impacts of nongenetic effects on milk yield traits in Holstein cattle.

Effects	Level	No. Records	DMY *	MY-Morn	MY-Noon	MY-Night
Parity	1	2541	29.86 ± 0.02 ^E^	9.98 ± 0.01 ^E^	10.06 ± 0.01 ^E^	9.83 ± 0.01 ^E^
	2	1005	34.72 ± 0.03 ^D^	11.61 ± 0.01 ^D^	11.68 ± 0.01 ^D^	11.42 ± 0.01 ^D^
	3	1311	35.95 ± 0.03 ^C^	12.04 ± 0.01 ^C^	12.06 ± 0.01 ^C^	12.85 ± 0.01 ^C^
	4	302	37.48 ± 0.06 ^A^	12.51 ± 0.02 ^A^	12.63 ± 0.02 ^A^	12.35 ± 0.02 ^A^
	5 and above	127	36.22 ± 0.07 ^B^	12.12 ± 0.03 ^B^	12.15 ± 0.03 ^B^	11.96 ± 0.03 ^B^
Lactation stage	1~44 d	3208	38.55 ± 0.04 ^B^	12.98 ± 0.02 ^B^	12.70 ± 0.02 ^B^	12.87 ± 0.02 ^B^
	45~99 d	3288	41.56 ± 0.03 ^A^	14.05 ± 0.01 ^A^	14.79 ± 0.01 ^A^	13.72 ± 0.01 ^A^
	100~199 d	3587	36.33 ± 0.03 ^C^	12.11 ± 0.01 ^C^	12.31 ± 0.01 ^C^	11.91 ± 0.01 ^C^
	200~305 d	3160	30.58 ± 0.03 ^D^	10.09 ± 0.01 ^D^	10.48 ± 0.01 ^D^	10.00 ± 0.01 ^D^
	>305 d	1246	27.22 ± 0.05 ^E^	9.03 ± 0.02 ^E^	9.28 ± 0.02 ^E^	8.91 ± 0.02 ^E^
Test season	Spring	717	36.16 ± 0.03 ^B^	12.12 ± 0.01 ^B^	12.20 ± 0.01 ^B^	11.84 ± 0.01 ^B^
	Summer	1378	31.58 ± 0.04 ^D^	10.58 ± 0.01 ^D^	10.53 ± 0.01 ^D^	10.47 ± 0.01 ^D^
	Fall	1271	33.96 ± 0.04 ^C^	11.39 ± 0.02 ^C^	11.34 ± 0.02 ^C^	11.23 ± 0.01 ^C^
	Winter	1451	37.69 ± 0.03 ^A^	12.52 ± 0.01 ^A^	12.78 ± 0.01 ^A^	12.39 ± 0.01 ^A^

* DMY, total milk yield in a day; MY-Morn, milk yield in the morning milking shift; MY-Noon, milk yield in the noon milking shift; MY-Night, milk yield in the night milking shift. In the same column of the same factor, values without the same capital letters mean significant difference (*p* < 0.01), while the same capital letter means no significant difference (*p* > 0.01).

**Table 4:** Impacts of nongenetic effects on milk production rate traits in Holstein cattle.

Effects	Level	No. Records	DMPR *	MPR-Morn	MPR-Noon	MPR-Night
Parity	1	2541	1.24 ^E^	1.25 ^E^	1.26 ^E^	1.23 ^E^
	2	1005	1.45 ^D^	1.45 ^D^	1.46 ^D^	1.43 ^D^
	3	1311	1.50 ^C^	1.50 ^C^	1.51 ^C^	1.48 ^C^
	4	302	1.56 ^A^	1.56 ^A^	1.58 ^A^	1.54 ^A^
	5 and above	127	1.51 ^B^	1.52 ^B^	1.52 ^B^	1.49 ^B^
Lactation stage	1~44 d	3208	1.61 ^B^	1.62 ^B^	1.59 ^B^	1.61 ^B^
	45~99 d	3288	1.73 ^A^	1.76 ^A^	1.73 ^A^	1.71 ^A^
	100~199 d	3587	1.51 ^C^	1.52 ^C^	1.54 ^C^	1.49 ^C^
	200~305 d	3160	1.27 ^D^	1.26 ^D^	1.31 ^D^	1.25 ^D^
	>305 d	1246	1.13 ^E^	1.13 ^E^	1.16 ^E^	1.11 ^E^
Test season	Spring	717	1.51 ^B^	1.52 ^B^	1.52 ^B^	1.48 ^B^
	Summer	1378	1.32 ^D^	1.32 ^D^	1.32 ^D^	1.31 ^D^
	Fall	1271	1.41 ^C^	1.42 ^C^	1.42 ^C^	1.41 ^C^
	Winter	1451	1.57 ^A^	1.58 ^A^	1.61 ^A^	1.54 ^A^

* DMPR, daily milk production rate; MPR-Morn, milk production rate in morning shift; MPR-Noon, milk production rate in noon shift; MPR-Night, milk production rate in night shift. In the same column of the same factor, values without the same capital letters mean significant difference (*p* < 0.01), while the same capital letter means no significant difference (*p* > 0.01). The standard errors (SE) for all least squares means in this table are less than 0.01; therefore, they are not displayed.

**Table 5:** Impacts of nongenetic effects on stability of milk production rate traits in Holstein cattle.

Effects	Level	No. Records	MPR-SD *	MPR-R
Parity	1	2541	0.1415 ± 0.0004 ^D^	0.3257 ± 0.0008 ^D^
	2	1005	0.1640 ± 0.0006 ^C^	0.3792 ± 0.0013 ^C^
	3	1311	0.1740 ± 0.0005 ^B^	0.4027 ± 0.0011 ^B^
	4	302	0.1810 ± 0.0011 ^A^	0.4194 ± 0.0025 ^A^
	5 and above	127	0.1788 ± 0.0013 ^A^	0.4143 ± 0.0030 ^A^
Lactation stage	1~44 d	3208	0.1884 ± 0.0007 ^B^	0.4336 ± 0.0016 ^B^
	45~99 d	3288	0.1919 ± 0.0006 ^A^	0.4435 ± 0.0014 ^A^
	100~199 d	3587	0.1667 ± 0.0005 ^C^	0.3865 ± 0.0012 ^C^
	200~305 d	3160	0.1433 ± 0.0006 ^E^	0.3324 ± 0.0013 ^E^
	>305 d	1246	0.1491 ± 0.0010 ^D^	0.3452 ± 0.0023 ^D^
Test season	Spring	717	0.1579 ± 0.0006 ^B^	0.3650 ± 0.0013 ^B^
	Summer	1378	0.2138 ± 0.0006 ^A^	0.4963 ± 0.0015 ^A^
	Fall	1271	0.1466 ± 0.0007 ^D^	0.3378 ± 0.0016 ^D^
	Winter	1451	0.1534 ± 0.0006 ^C^	0.3539 ± 0.0013 ^C^

* MPR-SD, standard deviation of milk production rate among three shifts per day; MPR-R, range of milk production rate among three shifts per day. In the same column of the same factor, values without the same capital letters mean extremely significant difference (*p* < 0.01), while the same capital letter means no extremely significant difference (*p* > 0.01).

**Table 6:** Estimates of additive genetic variance (σa2), permanent environmental variance(σpe2), residual variance (σe2) and heritability (h2) of indicators related to milk yield, milk production rate and its stability in Chinese Holsteins.

Trait	No. Records	σ_a_^2^	σ_pe_^2^	σ_e_^2^	h^2^ ± SE	Repeatability
DMY *	533,845	39.21	26.51	35.91	0.39 ± 0.02	0.65
MY-Morn	533,845	3.95	2.88	7.13	0.28 ± 0.02	0.49
MY-Noon	533,845	3.23	2.86	6.99	0.25 ± 0.02	0.47
MY-Night	533,845	3.52	2.78	6.73	0.27 ± 0.02	0.48
DMPR	533,845	0.07	0.05	0.06	0.39 ± 0.02	0.67
MPR-Morn	533,845	0.05	0.04	0.12	0.25 ± 0.02	0.43
MPR-Noon	533,845	0.05	0.04	0.11	0.25 ± 0.02	0.45
MPR-Night	533,845	0.06	0.04	0.10	0.30 ± 0.02	0.50
MPR-SD	533,845	<0.01	<0.01	0.02	0.04 ± 0.00	0.04
MPR-R	533,845	0.01	<0.01	0.11	0.05 ± 0.00	0.05

* DMY, total milk yield in a day; MY-Morn, milk yield in the morning milking shift; MY-Noon, milk yield in the noon milking shift; MY-Night, milk yield in the night milking shift; DMPR, daily milk production rate; MPR-Morn, milk production rate in the morning shift; MPR-Noon, milk production rate in the noon shift; MPR-Night, milk production rate in the night shift; MPR-SD, standard deviation of milk production rate among three shifts per day; MPR-R, range of milk production rate among three shifts per day.

**Table 7:** Estimates of genetic correlations of the stability of milk secretion rate with milk secretion rate and milk yield traits in Holsteins.

Trait	MSR-SD	MSR-R
DMY *	0.712 ± 0.034	0.717 ± 0.034
MY-Morn	0.706 ± 0.035	0.711 ± 0.034
MY-Noon	0.678 ± 0.039	0.683 ± 0.038
MY-Night	0.706 ± 0.036	0.711 ± 0.035
DMSR	0.713 ± 0.034	0.717 ± 0.033
MSR-Morn	0.686 ± 0.038	0.691 ± 0.037
MSR-Noon	0.679 ± 0.039	0.684 ± 0.038
MSR-Night	0.721 ± 0.034	0.726 ± 0.033

* DMY, total milk yield in a day; MY-Morn, milk yield in the morning milking shift; MY-Noon, milk yield in the noon milking shift; MY-Night, milk yield in the night milking shift; DMPR, daily milk production rate; MPR-Morn, milk production rate in the morning shift; MPR-Noon, milk production rate in the noon shift; MPR-Night, milk production rate in the night shift; MPR-SD, standard deviation of milk production rate among three shifts per day; MPR-R, range of milk production rate among three shifts per day.

## Data Availability

Restrictions apply to the availability of these data. Data were obtained from commercial dairy farm and are available from the author Yongjiang Mao with the permission of source farm.
